# Socioeconomic position and the health gradient in Cuba: dimensions and mechanisms

**DOI:** 10.1186/s12889-020-08980-3

**Published:** 2020-06-05

**Authors:** Peng Nie, Lanlin Ding, Alfonso Sousa-Poza, Alina Alfonso Leon, Hong Xue, Peng Jia, Liang Wang, Maria Elena Díaz Sánchez, Youfa Wang

**Affiliations:** 1 School of Economics and Finance, Xi’an Jiaotong University, Xi’an, 710061 China; 2 Institute for Health Care & Public Management, University of Hohenheim, Stuttgart, Germany; 3 Global Health Institute, Department of Epidemiology and Biostatistics, School of Public Health, Xi’an Jiaotong University, Xi’an, 710061 Shaanxi China; 4 Centre for Demographic Studies (CEDEM), University of Havana, Havana, Cuba; 5 Department of Health Administration and Policy, College of Health and Human Services, George Mason University, Fairfax, VA 22030 USA; 6 Department of Land Surveying and Geo-Informatics, The Hong Kong Polytechnic University, Hong Kong, China; 7 International Initiative on Spatial Lifecourse Epidemiology (ISLE), Hong Kong, China; 8 Department of Biostatistics and Epidemiology, East Tennessee State University, Johnson City, USA; 9 Instituto Nacional de Higiene de los Alimentos, Havana, Cuba

**Keywords:** Self-reported health, Biomarkers, Socioeconomic position, Cuba

## Abstract

**Background:**

To throw light on the under-researched association between socioeconomic position (SEP) and health in Cuba, this study examined SEP gradients in health and their underlying mechanisms among urban Cuban adults aged 18–65.

**Methods:**

By applying linear regressions to data from the 2010 National Survey on Risk Factors and Chronic Diseases, the analysis explored the SEP-health gradient along three SEP dimensions − education, occupation, and skin colour − using ten health measures: self-reported health (SRH), general and abdominal obesity, hypertension, high glucose, high cholesterol, high triglycerides, low high-density lipoprotein cholesterol, metabolic syndrome, and cumulative risk factors. Regressions also included behaviours and health-related risk perceptions (tobacco and alcohol consumption, diet, physical activity, and risk-related behaviours). It thus investigated the SEP-health gradient and its underlying mechanisms via both behaviours and health-related risk perceptions.

**Results:**

Once controlling for gender, age, marital status, region and provincial dummies, the analysis detected educational gradients in SRH (estimated coefficient [95% CI]: middle-level education = 3.535 [1.329, 5.741], *p* < 0.01; high-level education = 5.249 [3.050, 7.448], *p* < 0.01) that are partially explainable by both health-affecting behaviours (tobacco and alcohol consumption, diet, physical and sedentary activity) and risk perceptions. Using objective measures of health, however, it found no SEP-health gradients other than hypertension among people identified as having Black skin color (adjusted for demographic variables, 0.060 [0.018, 0.101], *p* < 0.01) and high cholesterol among those identified as having Mulatto or Mestizo skin color (adjusted for demographic variables, − 0.066 [− 0.098, − 0.033], *p* < 0.01).

**Conclusions:**

In terms of objective health measures, the study provides minimal evidence for an SEP-health gradient in Cuba, results primarily attributable to the country’s universal healthcare system − which offers full coverage and access and affordable medications − and its highly developed education system.

## Background

Although the social and medical sciences have largely established a positive relation (i.e., gradient) between socioeconomic position (SEP) and health in Europe [1–3], the US [4–8], and other high-income countries [9–12], the evidence in low and middle-income countries (LMICs) is less clear, with some studies identifying positive SEP-health gradients [13–15] whilst some showing none [16–19]. One possible reason for this inconsistency in the results in LMICs is that socioeconomic disparities within nations vary substantially among countries. At the same time, despite much effort expended on identifying the SEP-health gradient, its underlying mechanisms remain unclear. Yet in order to devise efficient policies to reduce health inequalities across different SEP dimensions, policy-makers need to fully understand the nature of this gradient and its underlying pathways.

Cuba offers a particularly interesting case for assessing the SEP-health gradient because despite stable economic growth and an egalitarian healthcare system, it has a rapidly ageing population and a substantial burden of noncommunicable chronic diseases (NCDs). One concern is that its near tripling of per capita gross domestic product (GDP) during 2001–2010 [20] was accompanied by an increase in income inequality that does not appear to be a function of educational inequality [21]. In addition, although its egalitarian healthcare system offers high capacity primary care, universal coverage and access, affordable medications, and robust clinical registries [22], the nation is experiencing an increase in NCDs [23], with an overall burden of blood pressure-related premature deaths of around 40% [22]. At the same time, the nation is ageing so rapidly that by 2020, 25% of its total population is likely to be 60 and older [24].

This study thus uses a combination of self-reported health (SRH), blood-based biomarkers, health-related behaviours, and risk perceptions to determine the presence of SEP-health gradients in Cuba and explore the mechanisms that may underlie the SEP impact on health. To ensure an objective assessment of disease risk, it employs both subjective and objective health measures (biomarkers), and adds the SEP dimensions of occupation and skin colour to the commonly used factors of income and education.

## Methods

### Study design and data

The data are taken from the 2010 National Survey on Risk Factors and Chronic Diseases (NSRFCD), administered collaboratively by Cuba’s National Institute of Hygiene, Epidemiology and Microbiology; National Statistics Bureau, and Nutrition and Food Hygiene Institute [25]. This nationally representative survey, which maintained all samples under strict quality control, applied a stratified multistage cluster sampling design covering 14 of Cuba’s provinces and the Isle of Youth special municipality. Although the survey was administered in all urban areas in 1995 (NSRFCD I) and 2001 (NSRFCD II) and in both urban and rural areas in 2010 (NSRFCD III), only the 2010 wave collected blood biomarker data and was thus used as the basis for present study. A total of 7915 individuals aged 15+ were surveyed in 2010.

### Health outcome measures

To combine the advantages of subjective and objective health indicators, we included SRH, individual biomarkers and composite measures of cardiometabolic disease/metabolic function. As the main measure of subjective health we used SRH, which encompasses not only mental and physical health but subjective experience of acute and chronic diseases and overall feelings of well-being [26]. SRH is generally considered a good predictor of mortality [27]. Our SRH measure is based on the following item: “To help people describe how good or bad their state of health is, we have drawn a scale similar to a thermometer, which is marked with a 100 for the best state of health that can be imagined and with a 0 for the worst state of health that you can imagine. We would like you to indicate on this scale with an arrow, in your opinion, how good or bad your health status is today”. Regarding objective health, we introduced physical measurements and blood-based biomarkers that directly relate to major chronic conditions such as general obesity, central obesity, high blood pressure, diabetes and coronary heart diseases. Specifically, the individual biomarker variables included body mass index (BMI), waist circumference (WC), blood pressure, total cholesterol, high-density lipoprotein (HDL) cholesterol and triglycerides. The two composite indicators included metabolic syndrome and cumulative risk factors.

Our binary health measures are better suited than continuous measures to a capture health state when both high and low values of a biomarkers as associated with a health risk [28]. Furthermore, all the markers in our analysis have well-established cutoff points denoting clinical risks. Finally, binary measures are robust to outliers which is particularly relevant when sample size are relatively small [28]. In our analysis, biomarker values were categorized into binary ones using cut-off points recommended by the International Diabetes Federation (IDF) or in other published studies [13, 18, 29, 30], with separate cut-off points for men and women where appropriate (see Table 1). In particular, metabolic syndrome was defined as having high abdominal girth and at least two of the following risk factors: diabetes, high triglycerides, low HDL cholesterol, and hypertension [18]. It was coded in a binary variable having a value equal to 1 if metabolic syndrome was present; 0 otherwise. Cumulative risk factors was coded in a binary variable equal to 1 if at least one cardiovascular disease risk factor was present (high waist circumference, diabetes, hypertension, high triglycerides, and high cholesterol ratio); 0 otherwise. Table 1 provides the cut-off points for each individual biomarker and definitions of cardiometabolic risk.

**Table 1:** Health indicators and definition of cardiometabolic risk, NSRFCD 2010

Health outcomes	Risk indicator	Definition
Body mass index (BMI, kg/m^2^)	General obesity	BMI ≥ 30 kg/m^2^
Waist circumference (WC, cm)	Central obesity	WC ≥ 90 cm for males; WC ≥ 80 cm for females
Blood pressure (mmHg)	Hypertension	Systolic blood pressure ≥ 140 mmHg or diastolic blood pressure ≥ 90 mmHg
Fasting blood glucose (mmol/l)	High glucose (Diabetes)	≥ 7 mmol/l
Total cholesterol (mmol/l)	High total cholesterol	≥ 6.22 mmol/l
HDL cholesterol (mmol/l)	Low HDL cholesterol	< 1.04 mmol/l
Triglycerides (mmol/l)	High triglycerides	≥ 2.26 mmol/l
Metabolic syndrome		Having high abdominal girth and at least two of the following risk factors: diabetes, high triglycerides, low HDL cholesterol, and hypertension.
CVD risk factors		A tally of cardiovascular disease risk factors including high waist circumference, diabetes, hypertension, high triglycerides, and high cholesterol ratio (calculated as the ratio of total cholesterol to HDL cholesterol)

Sources: Rosero-Bixby & Dow and Goldman [27]

### SEP measures

Since a variety of socioeconomic variables, including education, income, and occupation, along with self-assessed skin color, among others, show similar associations with health, a broader underlying dimension of social stratification or social ordering is needed when assessing the relation between SEP and health. Furthermore, different SEP measures may have differing effects on certain health outcomes [31]. Thus, existing studies on this topic put great emphasis on the multidimensional nature of SEP [31–33]. In accordance with this literature, our paper captures three important SEP dimensions, namely education, occupation and skin colour. Specifically, education is measured on a 3-point scale (low: illiterate/primary school; medium: secondary school/qualified worker/technical school; high: high school/university), with two dummy variables for “high” versus “medium,” with “low” as the reference. In addition, occupation is measured on a 4-point scale (1: unemployed; 2: housewife/husband; 3: self-employed; 4: state worker) and recoded as a set of dummy variables with “unemployed” as the reference. Contrary to other SEP dimensions, there is less of an ordering in the occupational categories – especially in the Cuban context. For example, self-employment is a common form of private entrepreneurial activity in Cuba [34] and it provides a viable alternative to many former State sector employees who suffered job losses or have been temporarily place on paid leave due to state-owned enterprises’ restructuring [35]. Since 2009 the share of workers in the state sector has declined sharply whilst the non-state sectors’ share has increased dramatically. The rate of self-employed workers to total employed workers has increased from 2.83% in 2009 to 8.25% in 2012 [36]. In addition, Cuba’s official unemployment rate has been remarkably low in comparison to other economies, including all those in Latin America. However, as Hernández-Catá [37] have emphasized: “Given the peculiar characteristics of the Cuban economy, the conventionally defined unemployment rate is a very bad indicator of labor market conditions” (p. 2). This is attributable to the failure of taking disguised unemployment and discouraged workers into account [37]. Skin colour is measured on a 3-point scale (1: people identified as having White skin color; 2: people identified as having Mulatto or Mestizo skin colour; 3: people identified as having Black skin colour) and then converted into a set of dummy variables with “those identified as having White skin colour” as the reference. It is important to note that skin colour refers to an individual’s perception of his or her skin colour. Although skin colour identity can evolve within one individual, it is a characteristic less amenable to change than the other SEP measures.

### Additional SEP measure

Income is measured as monthly income in Cuban pesos (CUP). This indicator was included because, despite legitimate concerns about NSRFCD income data reliability (around 43% of missing values), it is widely used in evaluating SEP-health gradients. Besides the questionable reliability of the NSRFCD income data, such surveyed income measures have somewhat less relevance in the Cuban setting than in other countries, especially as relatively low nominal salaries are offset by subsidized healthcare, education, transportation and social services, and are supplemented by remittances from abroad [36]. Nevertheless, inflation, monetary dualism, gradual price liberalization (especially with regards to food and agricultural products), and the partial elimination of state subsidies have all contributed to the decline of the real purchasing power for most Cuban households [36].

### Behavioural and risk perception measures

One component of research on the SEP-health gradient is the mechanisms underlying this gradient, that is the behavioural characteristics –health-enhancing behaviours such as healthy diets and physical exercise or health-compromising activities such as smoking or drinking alcohol [13] – which may drive the association between SEP and health. For example, income shocks are detrimental to individual lifestyles that include smoking and social drinking [38–41]. A higher level of education can positively affect individual health status mainly because one of the likely benefits of higher education is general knowledge (and in particular medical knowledge) that helps individuals become more health-conscious and take preventive actions [42, 43]. In order to identify the underlying mechanisms through which the different dimensions of SEP operate on health, the analysis explored three types of health-affecting behaviours:

1. Tobacco and alcohol consumption: measured by responses to two survey questions: “Do you currently smoke any tobacco products, such as cigarettes, cigars or pipes?” (1: yes; 0: no); “Have you consumed any drinks that contain alcohol, such as beer, wine, moonshine, cider or others during the previous month?” (1: yes; 0: no).
2. Diet: measured by five indicators: (i) frequency of eating fruits and vegetables: “On a typical week, how many days do you eat fruits/vegetables?” (1: 0–2 days; 2: 3–4 days; 3: 5–7 days); (ii) meals away from home during a week: “On a typical week, how many meals do you have outside the home?” (times/week); (iii) type of cooking oil consumed: “What kind of oil do you usually use at home to prepare foods?” (1: vegetable oil; 0: other); (iv) eating breakfast: “Are you used to having breakfast?” (1: yes, every day; 2: not always; 3: no); (v) salt consumption: “Do you add salt to your meals after they are cooked?” (1: yes; 2: sometimes; 3: no).
3. Physical activity (PA) versus sedentary activity (SA): PA was measured by responses to three questions: “Does your job demand any kind of intensity physical activity that leads to an important acceleration of breathing or heart rate?” (1: yes; 0: no); “Do you walk or ride a bicycle for at least 10 consecutive minutes when going about” (1: yes; 0: no); “In your free time, do you practice any kind of moderate intensity activity that involves a light acceleration of breathing or heart rate?” (1: yes; 0: no). SA was measured by responses to one question: “How long do you spend sitting or lying back on a typical day?” (hours/day).

The study also assessed risk perceptions for eight such activities:

- Risk-related behaviours: (i) smoking, (ii) drinking alcohol occasionally, (iii) remaining where smokers are present even though you do not smoke, (iv) doing little physical exercise, (v) eating few fruits, (vi) adding salt to cooked food, (vii) eating few vegetables, (viii) being overweight, and (ix) eating pork fat.
- Measurement: respondent rankings along a 4-point scale: 1: no health risk; 2: little health risk; 3: moderate health risk; 4: substantial health risk:

### Covariates

As highlighted by Fuchs [44], health deteriorates with age, which is a fundamental fact of biology. Furthermore, gender difference in life expectancy has been discernable in almost every country [44]. In our health outcomes estimations we therefore introduced age and gender. In addition, given that marital status might affect household production of health caregiving and demand for health [44], we controlled for marital status in our analyses. To capture possible geographical heterogeneities in different health outcomes, we also introduced urban and provincial controls. Following these existing studies [31, 44, 45], our models included the following general demographics as analytic covariates: (i) gender (1: male; 0: female); (ii) age; (iii) marital status (three binary variables capturing singles, married/cohabiting individuals, divorced/separated/widowed individuals; with singles as the reference category); (iv) an urban dummy (1: urban; 0: rural); and (v) a provincial dummy, with Pinar del Río as the reference (see Table 1 for the descriptive statistics).

### Statistical analysis

Since we focused on different dimensions of cardiometabolic risks, we adopted dichotomous variables in our regression analysis, which have been used extensively in existing studies on identifying SEP-health gradients [13, 18, 29]. After first applying an ordinary least squares (OLS) model to assess the SEP-SRH gradient, the analysis used linear probability models (LPMs) to estimate the SEP-health gradients based on individual and composite biomarkers (all performed using STATA/SE version 14) [46]. LPMs were deemed the most suitable because, unlike nonlinear probit or logit models whose maximum likelihood estimations are inconsistent in the presence of heteroscedasticity [47], they provide consistent estimates despite heteroscedasticity of the error term, as well as easily interpretable coefficients. The models used to estimate the SEP-health gradients contained different covariate sets: Whereas model 1 included gender, age, marital status, region and provincial dummy variables, models 2 and 3 stepwise added in the three SEP dimensions and health-related behaviours, respectively. Model 4 then included all the model 1 variables plus risk perceptions, after which model 5 controlled for all the covariates (including gender, age, marital status, region, provincial dummy variables, tobacco and alcohol consumption, diet, PA, and risk-related behaviours) simultaneously. The samples were weighted to ensure nationally representative estimates.

## Results

### Study population characteristics

This analytic sample was restricted to adults aged 18–65 (SRH, *n* = 4124; general obesity, *n* = 3741; abdominal obesity, *n* = 3764; hypertension: 3757; high glucose, *n* = 1009; high total cholesterol, *n* = 1018, high triglycerides, *n* = 1008; low high-density lipoprotein cholesterol, *n* = 246; metabolic syndrome, *n* = 244 and cardiovascular disease (CVD) risk factor, *n* = 234) for which complete demographic, SEP, health-related behaviour, and biomarker information was available.

As shown in Table 2, the mean SRH was around 87. The average values of BMI and WC were 25.4 kg/m^2^ and 83.8 cm, respectively, and the rates of general obesity and abdominal obesity were 16.1 and 45.3%, respectively. For cardiometabolic risks, the prevalence rates of hypertension was 17.5%. Approximately 16% of Cubans have high total cholesterol. Interestingly, however, about 57% of Cubans have low levels of HDL cholesterol. Thus, although especially NCDs are on the rise in Cuba, the general picture one gets from these set of indicators is that the Cuban population is healthier (with the exception of low HDL cholesterol) than the American. As can be seen in Table 3, there is some variation in the SEP variables used in our analysis, especially with regards to education: 11% illiterate or primary school, 27% secondary school, 2% qualified worker, 19% technical school, 28% high school, and 14% university. Variation in occupation is substantially lower, with over 64% classified as state workers. Nevertheless, about 9% of the sample are self-employed. With regards to skin colour, being White was the majority (65.7%), compared with being Mulatto/Mestizo and Black, with proportions of 24.6 and 9.7%, respectively. Regarding health-related behaviours, approximately 25.5 and 53.2% of Cubans were smoke tobacco and consume alcohol, respectively (Table 4). 26.5 and 33.1% of Cubans ate fruits and vegetables 5–7 days per week, respectively. 90.0% of Cubans reported smoking and 55.6% reported drinking alcohol occasionally as a substantial health risk. However, merely 36.3 and 41.4% of Cubans considered eating few fruits and vegetables, respectively, as a substantial health risk.

**Table 2:** Health characteristics for Cuban aged 18–65, NSRFCD 2010

Health characteristics	Mean/proportion (%)	Std. Dev	N
Self-reported health (SRH, 0–100)	86.8	16.7	4124
Body mass index (BMI, kg/cm^2^)	25.4	5.2	3741
Waist circumference (cm)	83.8	12.6	3764
Systolic blood pressure (SBP, mmHg)	118.7	16.3	3757
Diastolic blood pressure (DBP, mmHg)	76.7	11.5	3757
Glucose (mmol/l)	4.8	1.8	1009
Total cholesterol (mmol/l)	7.8	11.0	1018
HDL cholesterol (mmol/l)	1.4	1.4	246
Triglycerides (mmol/l)	2.6	3.7	1008
General obesity (1 = Yes; 0 = No)	16.1		3741
Abdominal obesity (1 = Yes; 0 = No)	45.3		3764
Hypertension (1 = Yes; 0 = No)	17.5		3757
High glucose (1 = Yes; 0 = No)	4.9		1009
High total cholesterol (1 = Yes; 0 = No)	16.0		1018
Low HDL cholesterol (1 = Yes; 0 = No)	57.3		246
High triglycerides (1 = Yes; 0 = No)	21.1		1008
Metabolic syndrome (1 = Yes; 0 = No)	11.9		244
CVD risk factors	1.4	1.0	234

**Table 3:** Descriptive statistics for Cuban aged 18–65, NSRFCD 2010

Variables	Mean/proportion (%)	Std. Dev	N
Gender (1 = male; 0 = female)	47.8		5497
Age	40.7	11.9	5497
Region (1 = urban; 0 = rural)	73.1		5497
Marital status			5497
Single	24.0		1322
Married/living together	66.5		3655
Widowed/separated/divorced	9.5		520
Monthly income (log)	6.1	0.6	3127
Education			5497
No schooling	3.2		174
Primary school	8.2		449
Secondary school	26.7		1468
Qualified worker	1.8		99
Technical school	18.7		1028
High school	27.8		1526
University	13.7		753
Employment status			5479
Unemployed	5.8		320
Housewife/houseman	21.9		1200
Self-employed	8.7		477
State worker	63.6		3482
Skin colour			5497
White	65.7		3612
Mulatto or Mestizo	24.6		1351
Black	9.7		534

**Table 4:** Descriptive statistics of behavioral and risk perception measures

Variables	N	Mean/Proportion (%)
***Behaviours***		
**Tobacco and alcohol consumption**		
Tobacco (1 = yes; 0 = no)	5497	25.5
Alcohol (1 = yes; 0 = no)	5496	53.2
**Diets**		
Frequency of eating fruits (days/week)	5286	
0–2 days	2120	40.1
3–4 days	1764	33.4
5–7 days	1402	26.5
Frequency of eating vegetables (days/week)	5280	
0–2 days	1870	35.4
3–4 days	1662	31.5
5–7 days	1748	33.1
Meals away from home during a week (times/week)	5225	
0–2	3642	69.7
3–5	1000	19.1
≥ 6	583	11.2
Type of cooking oil consumed (1 = vegetable oil; 0 = others)	5490	86.5
Eating breakfast	5497	
Yes, everyday	3572	65.0
Not always	1035	18.8
No	890	16.2
Adding salt to meals after being cooked	5497	
Yes	276	5.0
Sometimes	489	8.9
No	4732	86.1
**PA**		
Intense physical activity during working (1 = yes; 0 = no)	5480	21.5
Riding bicycle or walking when going about (1 = yes; 0 = no)	5481	71.7
Moderate intensity activity when in free time (1 = yes; 0 = no)	5461	20.5
Sedentary time (hours/day)	5241	3.5
***Risk perception***		
Smoking	5242	
No health risk	287	5.5
Little health risk	45	0.9
Moderate health risk	191	3.6
Substantial health risk	4719	90.0
Drinking alcohol occasionally	5242	
No health risk	487	9.3
Little health risk	459	8.8
Moderate health risk	1382	26.4
Substantial health risk	2914	55.6
Staying places where there are smokers but you do not smoke	5240	
No health risk	266	5.1
Little health risk	175	3.3
Moderate health risk	960	18.3
Substantial health risk	3839	73.3
Doing little physical exercise	5240	
No health risk	532	10.2
Little health risk	592	11.3
Moderate health risk	1955	37.3
Substantial health risk	2161	41.2
Eating few fruits	5242	
No health risk	681	13.0
Little health risk	705	13.4
Moderate health risk	1954	37.3
Substantial health risk	1902	36.3
Adding salt to food after being cooked	5241	
No health risk	382	7.3
Little health risk	193	3.7
Moderate health risk	722	13.8
Substantial health risk	3944	75.3
Eating few vegetables	5242	
No health risk	562	10.7
Little health risk	599	11.4
Moderate health risk	1912	36.5
Substantial health risk	2169	41.4
Being overweight	5242	
No health risk	304	5.8
Little health risk	105	2.0
Moderate health risk	561	10.7
Substantial health risk	4272	81.5
Eating pork fat	5242	
No health risk	292	5.6
Little health risk	174	3.3
Moderate health risk	842	16.1
Substantial health risk	3934	75.0

### SEP and health gradient: the three dimensions

Table 5 shows the results of the SEP-health gradients with the adjustment of demographic characteristics. Table A1 provides a summary of associations between SEP and specific health outcomes. With general demographics and the three SEP dimensions controlled for (estimated coefficient [95% CI]: medium level education = 3.535 [1.329, 5.741], *p* < 0.01; high-level education = 5.249 [3.050, 7.448], *p* < 0.01), a better SRH was uniformly associated with a higher educational level. No such link emerged, however, between education and objective health outcomes. Both these latter and SRH also appeared unrelated to occupation, except in the case of general obesity, which was likely to be higher for a housewife or husband than for an unemployed worker. Whereas people that identified as having Black skin colour (relative to those identified as having White skin colour) was correlated with a higher probability of hypertension (0.060 [0.018, 0.101], *p* < 0.01), those identified as having Mulatto or Mestizo skin colour lowered the likelihood of high cholesterol (− 0.066, [− 0.098, − 0.033], *p* < 0.01). No associations were observable, however, between objective health outcomes and income (with general demographics controlled for, Table 5), although a better SRH did correlate with a higher monthly income (see Supplementary Materials Table A2 Panel A). We did not observe any income-health gradients when using both individual and composite objective health measures (see Supplementary Materials Tables A2-A4). Once general demographics were controlled for, all SEP dimensions remained uncorrelated with the two composite indicators of health outcomes, namely metabolic syndrome and cumulative risk factors.

**Table 5:** OLS and linear probability estimates for SEP-health gradients among Cuban aged 18–65: NSRFCD 2010 (with sociodemographics)

	SEP Dimension						
Health outcome	Education (Ref. = low)		Occupation (Ref. = unemployed)			Skin colour (Ref. = White)	
	Middle	High	Housewife/houseman	Self-employed	State worker	Mulatto/ Mestizo	Black
1. SRH	*N* = 4124						
Model 1	3.535*	5.249*	−0.536	1.695	1.886	1.091	− 0.051
	[1.329,5.741]	[3.050,7.448]	[−3.021,1.950]	[−1.646,5.037]	[− 0.716,4.489]	[− 0.299,2.480]	[−1.883,1.781]
Model 2	3.181	4.726*	−0.159	1.584.	1.332	1.112	− 0.132
	[0.825,5.536]	[2.455,6.997]	[−2.466,2.149]	[−1.637,4.806]	[− 1.221,3.885]	[− 0.305,2.528]	[− 1.968,1.704]
2. Hypertension	*N* = 3757						
Model 1	0.037	0.029	0.011	0.035	0.028	0.003	0.060*
	[−0.007,0.080]	[− 0.008,0.067]	[− 0.044,0.066]	[− 0.054,0.124]	[− 0.033,0.090]	[− 0.041,0.046]	[0.018,0.101]
Model 2	0.033	0.024	0.014	0.036	0.025	0.003	0.059*
	[−0.015,0.081]	[−0.020,0.068]	[− 0.041,0.068]	[− 0.052,0.125]	[− 0.038,0.088]	[− 0.040,0.047]	[0.017,0.101]
3. General obesity	*N* = 3741						
Model 1	−0.018	− 0.022	0.064	0.018	0.029	0.012	0.041
	[−0.055,0.019]	[−0.059,0.014]	[0.017,0.110]	[−0.026,0.062]	[− 0.005,0.062]	[− 0.019,0.043]	[− 0.029,0.112]
Model 2	−0.013	− 0.015	0.063	0.020	0.030	0.012	0.043
	[−0.052,0.026]	[−0.049,0.020]	[0.017,0.109]	[−0.023,0.063]	[− 0.005,0.065]	[− 0.019,0.043]	[− 0.027,0.112]
4. Abdominal obesity	*N* = 3764						
Model 1	− 0.036	− 0.006	0.003	− 0.012	0.011	0.015	0.013
	[−0.099,0.026]	[−0.076,0.065]	[− 0.113,0.118]	[− 0.121,0.097]	[− 0.084,0.106]	[− 0.038,0.068]	[− 0.055,0.082]
Model 2	− 0.040	− 0.011	0.003	−0.012	0.011	0.015	0.012
	[−0.106,0.026]	[−0.087,0.066]	[− 0.110,0.115]	[− 0.119,0.096]	[− 0.089,0.111]	[− 0.038,0.068]	[− 0.055,0.079]
5. High glucose	*N* = 1009						
Model 1	−0.024	− 0.021	0.052	0.030	0.036	−0.013	0.009
	[−0.086,0.039]	[−0.086,0.044]	[0.005,0.100]	[−0.013,0.072]	[0.005,0.067]	[−0.044,0.017]	[− 0.032,0.051]
Model 2	−0.023	− 0.020	0.051	0.030	0.038	−0.013	0.008
	[−0.084,0.037]	[−0.077,0.037]	[0.003,0.099]	[−0.015,0.074]	[0.009,0.066]	[−0.045,0.018]	[− 0.033,0.049]
6. High cholesterol	*N* = 1018						
Model 1	−0.007	−0.010	− 0.034	−0.028	− 0.037	−0.066*	0.040
	[−0.084,0.071]	[−0.094,0.073]	[− 0.104,0.037]	[− 0.157,0.100]	[−0.146,0.073]	[− 0.098,-0.033]	[− 0.083,0.163]
Model 2	− 0.002	−0.007	− 0.038	−0.032	− 0.042	−0.066*	0.042
	[−0.067,0.063]	[−0.089,0.075]	[− 0.123,0.047]	[− 0.169,0.105]	[−0.162,0.078]	[− 0.097,-0.035]	[− 0.082,0.166]
7. High triglycerides	*N* = 1008						
Model 1	0.017	−0.032	0.052	−0.022	−0.005	− 0.023	−0.078
	[−0.109,0.143]	[−0.145,0.082]	[− 0.091,0.194]	[− 0.179,0.136]	[− 0.130,0.121]	[− 0.090,0.043]	[− 0.163,0.007]
Model 2	0.027	−0.015	0.055	−0.019	0.003	−0.028	− 0.080
	[−0.092,0.147]	[− 0.120,0.091]	[− 0.091,0.200]	[− 0.182,0.144]	[− 0.123,0.130]	[− 0.093,0.038]	[−0.163,0.003]
8. Low HDL cholesterol	*N* = 246						
Model 1	0.076	0.135	−0.034	0.085	0.086	0.065	0.134
	[−0.108,0.261]	[−0.077,0.346]	[− 0.166,0.098]	[− 0.431,0.600]	[0.035,0.137]	[− 0.063,0.192]	[−0.074,0.343]
Model 2	0.052	0.077	−0.047	0.075	0.071	0.076	0.133
	[−0.218,0.323]	[−0.227,0.382]	[− 0.193,0.099]	[− 0.570,0.720]	[− 0.002,0.144]	[− 0.042,0.194]	[− 0.053,0.318]
9. Metabolic syndrome	*N* = 244						
Model 1	− 0.040	−0.011	0.106	−0.022	0.090	0.029	0.063
	[−0.299,0.219]	[−0.280,0.259]	[− 0.118,0.331]	[− 0.258,0.213]	[0.003,0.176]	[− 0.097,0.155]	[− 0.006,0.131]
Model 2	−0.055	− 0.026	0.095	−0.042	0.076	0.029	0.060
	[−0.240,0.130]	[−0.199,0.146]	[− 0.103,0.293]	[− 0.229,0.145]	[− 0.017,0.169]	[− 0.087,0.145]	[0.003,0.116]
10. One CVD risk factor	*N* = 234						
Model 1	−0.047	−0.007	− 0.032	0.103	0.025	0.047	0.024
	[− 0.166,0.072]	[−0.063,0.048]	[− 0.622,0.558]	[− 0.360,0.566]	[− 0.534,0.584]	[− 0.086,0.179]	[− 0.106,0.153]
Model 2	− 0.051	− 0.027	− 0.039	0.094	0.020	0.056	0.027
	[−0.177,0.075]	[−0.088,0.033]	[− 0.640,0.563]	[− 0.376,0.563]	[− 0.570,0.610]	[− 0.096,0.207]	[− 0.137,0.192]

Notes: Sample weights are applied. 95% confidence intervals in brackets

Model 1: OLS/LPM regressions adjusted for gender, age, marital status, region and provincial dummies

Model 2: Model 1 (Table 5) + other SEPs

* *p* < 0.01

### SEP and the health gradient: underlying mechanisms

The exploration of mechanisms underlying SEP’s impact on health included individual behaviours (tobacco and alcohol consumption, diet, physical and sedentary activities) and risk perceptions (see Table 6). One notable outcome was that although the SRH gradient in high-level education became flatter once behavioural variables were controlled for, the addition of health-related risk perceptions had little effect on the gradient. In addition, although controlling for health-affecting behaviours, risk perceptions, or even both together diminished the hypertension differences regarding people identified as having Black skin colour, the addition of these behaviours partially accounted for the differences in high total cholesterol (especially for those identified as having Mulatto or Mestizo skin colour), which health-related risk perceptions did not.

**Table 6:** OLS and linear probability estimates for underlying mechanisms of SEP-health gradients among Cuban aged 18–65: NSRFCD 2010 (with behavioural and risk perceptions)

Health outcome	SEP Dimension						
	Education (Ref. = low)		Occupation (Ref. = unemployed)			Skin colour (Ref. = White)	
	Middle	High	Housewife/houseman	Self-employed	State worker	Mulatto/ Mestizo	Black
1. SRH	*N* = 4124						
Model 3	3.364*.	4.943*	−0.967	1.253	1.477	1.109	0.175
	[1.340,5.387]	[2.945,6.941]	[−3.437,1.503]	[−1.939,4.445]	[−1.225,4.179]	[−0.189,2.406]	[− 1.651,2.002]
Model 4	3.525*	5.094*.	−0.156.	1.738	2.120	1.175	0.115
	[1.399,5.652]	[3.151,7.036]	[−2.347,2.036]	[− 1.587,5.062]	[− 0.438,4.679]	[− 0.112,2.461]	[− 1.711,1.941]
Model 5	3.030	4.282*	− 0.253	1.194.	1.296.	1.169	0.250
	[0.860,5.200]	[2.346,6.218]	[−2.366,1.859]	[−1.902,4.290]	[−1.395,3.988]	[− 0.044,2.383]	[− 1.529,2.029]
2. Hypertension	*N* = 3757						
Model 3	0.034	0.027	0.008	0.025	0.023	0.005	0.059
	[−0.013,0.080]	[−0.012,0.067]	[− 0.048,0.064]	[− 0.068,0.118]	[− 0.042,0.087]	[− 0.039,0.049]	[0.016,0.102]
Model 4	0.042	0.034	0.015	0.037	0.030	0.004	0.060
	[−0.003,0.086]	[−0.004,0.073]	[− 0.048,0.078]	[− 0.058,0.132]	[− 0.039,0.099]	[− 0.041,0.049]	[0.017,0.104]
Model 5	0.036	0.030	0.016	0.029	0.021	0.006	0.059
	[−0.013,0.086]	[−0.014,0.074]	[− 0.047,0.078]	[− 0.069,0.127]	[− 0.052,0.094]	[− 0.039,0.051]	[0.014,0.103]
3. General obesity	*N* = 3741						
Model 3	−0.024	− 0.030	0.064	0.011	0.020	0.015	0.043
	[−0.061,0.012]	[−0.062,0.002]	[0.017,0.110]	[−0.037,0.059]	[− 0.011,0.052]	[− 0.018,0.048]	[− 0.027,0.113]
Model 4	− 0.011	− 0.018	0.069	0.022	0.032	0.014	0.043
	[−0.053,0.031]	[−0.058,0.021]	[0.019,0.118]	[−0.021,0.066]	[− 0.004,0.069]	[− 0.015,0.043]	[− 0.023,0.109]
Model 5	− 0.010	− 0.016	0.069*	0.016	0.024	0.017	0.046
	[− 0.051,0.031]	[−0.051,0.019]	[0.020,0.117]	[−0.031,0.064]	[− 0.011,0.059]	[− 0.014,0.049]	[− 0.019,0.111]
4. Abdominal obesity	*N* = 3764						
Model 3	−0.053	− 0.030	− 0.001	− 0.018	− 0.006	0.024	0.025
	[− 0.121,0.016]	[−0.105,0.044]	[− 0.115,0.113]	[− 0.138,0.102]	[− 0.108,0.097]	[− 0.030,0.079]	[− 0.044,0.095]
Model 4	− 0.034	− 0.009	0.002	− 0.017	0.006	0.024	0.018
	[−0.095,0.027]	[−0.078,0.060]	[− 0.116,0.120]	[− 0.123,0.089]	[− 0.092,0.103]	[− 0.028,0.076]	[− 0.055,0.092]
Model 5	− 0.048	− 0.031	− 0.002	− 0.021	− 0.008	0.032	0.029
	[− 0.116,0.020]	[− 0.109,0.047]	[− 0.115,0.111]	[− 0.136,0.094]	[− 0.120,0.104]	[− 0.021,0.085]	[− 0.044,0.103]
5. High glucose	*N* = 1009						
Model 3	− 0.022	− 0.020	0.056	0.034	0.037	−0.014	0.016
	[−0.079,0.036]	[− 0.074,0.034]	[0.001,0.111]	[−0.025,0.092]	[− 0.008,0.083]	[− 0.045,0.016]	[− 0.026,0.058]
Model 4	− 0.022	− 0.022	0.065	0.038	0.045*	−0.009	0.007
	[−0.080,0.035]	[−0.086,0.041]	[0.012,0.118]	[−0.013,0.090]	[0.014,0.075]	[−0.038,0.020]	[− 0.052,0.066]
Model 5	−0.020	− 0.020	0.064	0.040	0.045	−0.011	0.010
	[−0.075,0.036]	[−0.068,0.029]	[0.008,0.121]	[−0.026,0.107]	[0.010,0.080]	[−0.039,0.018]	[− 0.046,0.067]
6. High cholesterol	*N* = 1018						
Model 3	−0.013	−0.013	− 0.044	−0.036	− 0.033	− 0.063*	0.040
	[−0.077,0.050]	[− 0.085,0.059]	[− 0.114,0.025]	[− 0.156,0.083]	[− 0.130,0.064]	[− 0.094,-0.031]	[− 0.081,0.161]
Model 4	− 0.007	− 0.021	− 0.050	− 0.036	− 0.052	− 0.065*	0.025
	[−0.099,0.085]	[− 0.117,0.074]	[− 0.123,0.023]	[− 0.168,0.096]	[− 0.176,0.072]	[− 0.095,-0.035]	[− 0.082,0.132]
Model 5	− 0.014	− 0.030	− 0.066	− 0.048	− 0.049	− 0.062*	0.029
	[− 0.084,0.056]	[− 0.116,0.056]	[− 0.153,0.021]	[− 0.182,0.085]	[− 0.171,0.072]	[− 0.092,-0.032]	[− 0.080,0.139]
7. High triglycerides	*N* = 1008						
Model 3	0.023	− 0.023	0.047	− 0.025	0.008	− 0.023	− 0.071
	[− 0.096,0.143]	[− 0.144,0.097]	[− 0.088,0.181]	[− 0.170,0.121]	[− 0.114,0.129]	[− 0.085,0.039]	[− 0.138,-0.003]
Model 4	− 0.009	− 0.058	0.028	−0.020	− 0.014	− 0.020	− 0.091
	[−0.130,0.112]	[− 0.169,0.052]	[− 0.151,0.207]	[−0.191,0.152]	[− 0.167,0.140]	[− 0.081,0.042]	[−0.178,-0.004]
Model 5	0.001	−0.045	0.023	−0.020	0.007	−0.020	−0.084
	[−0.107,0.109]	[−0.156,0.066]	[− 0.150,0.196]	[− 0.192,0.152]	[−0.146,0.160]	[− 0.078,0.037]	[− 0.153,-0.015]
8. Low HDL cholesterol	*N* = 246						
Model 3	0.079	0.164	−0.063	0.094	0.044	0.061	0.111
	[−0.251,0.408]	[−0.226,0.554]	[− 0.167,0.041]	[− 0.212,0.400]	[−0.436,0.524]	[0.005,0.117]	[−0.024,0.246]
Model 4	0.120	0.218	−0.132	0.042	0.025	0.098	0.149
	[−0.181,0.420]	[−0.168,0.604]	[− 0.437,0.172]	[− 0.766,0.850]	[−0.391,0.442]	[0.013,0.183]	[−0.190,0.487]
Model 5	0.155	0.272	−0.169	0.004	−0.067	0.097	0.088
	[−0.453,0.763]	[−0.394,0.938]	[− 0.355,0.018]	[− 0.584,0.591]	[−0.438,0.304]	[− 0.042,0.236]	[− 0.137,0.314]
9. Metabolic syndrome	*N* = 244						
Model 3	0.001	0.019	0.080	−0.076	0.046	0.031	0.057
	[−0.341,0.342]	[−0.334,0.371]	[− 0.209,0.368]	[− 0.446,0.294]	[−0.194,0.286]	[− 0.103,0.166]	[0.001,0.113]
Model 4	−0.023	−0.0002	0.080	0.002	0.069	0.012	0.030
	[−0.231,0.184]	[−0.218,0.218]	[− 0.044,0.204]	[− 0.246,0.250]	[0.002,0.136]	[− 0.082,0.106]	[−0.097,0.158]
Model 5	0.002	0.038	0.041	−0.073	0.030	0.014	0.022
	[−0.278,0.282]	[−0.244,0.321]	[− 0.162,0.245]	[− 0.502,0.357]	[−0.155,0.214]	[− 0.095,0.123]	[− 0.034,0.079]
10. One CVD risk factor	*N* = 234						
Model 3	−0.072	−0.043	− 0.072	0.070	− 0.031	0.064	0.008
	[−0.396,0.253]	[−0.233,0.147]	[− 0.549,0.405]	[− 0.247,0.388]	[−0.550,0.488]	[0.017,0.112]	[−0.206,0.223]
Model 4	−0.048	0.050	−0.145	−0.033	− 0.038	0.040	− 0.088
	[−0.228,0.133]	[− 0.026,0.125]	[− 0.787,0.497]	[−0.549,0.483]	[− 0.569,0.493]	[− 0.092,0.172]	[−0.161,-0.016]
Model 5	−0.061	0.007	−0.158	−0.048	− 0.070	0.061	− 0.098
	[−0.500,0.379]	[− 0.356,0.370]	[− 0.518,0.201]	[−0.167,0.070]	[− 0.434,0.293]	[− 0.011,0.133]	[−0.152,-0.043]

Notes: Sample weights are applied. 95% confidence intervals in brackets

Model 3: Model 1 (Table 5) + behavioural variables

Model 4: Model 1 (Table 5) + risk perceptions

Model 5: Model 1 (Table 5) + other SEPs + behavioural variables + risk perceptions

* *p* < 0.01

## Discussion

This study was a first attempt to combine both subjective and objective health measures to assess the SEP-health gradient in Cuba and identify its underlying mechanisms. Although the analysis confirmed the presence of education and income gradients in SRH − both partially explainable by health-affecting behaviours and risk perceptions − the use of objective health measures generally failed to identify any SEP-health gradients except for differences based on skin colour in hypertension and high cholesterol.

The analysis did, however, reveal significant discrepancies in the SEP-health gradient depending on whether the health measures used were subjective or objective. Hence, although SRH is acknowledged to be a good predictor of mortality following medical care [42], a certain amount of caution is warranted when using subjective measures to determine the SEP-health gradient. On the other hand, the education/income-SRH gradients identified resemble those observed in other high-income countries [2, 48, 49], and their mediation by health-affecting behaviours is mirrored in other Western studies [38, 39], indicating that the SEP-health gradient is partially attributable to smoking and social drinking. In one Costa Rican study, for example, using SRH as a measure indicated the presence of an SEP-health gradient but employing CVD risk factors like diabetes and total cholesterol did not [18]. Similarly, in a Mexican study, the SEP-health gradients identified using SRH vanished with the use of objective health indicators like obesity [29]. A study for China even documented that although higher SEPs were associated with better SRH [30], they were uncorrelated with objective health measures like obesity and hypertension [16].

Interestingly, the rates of general obesity and abdominal obesity (15.4 and 44.1%) were quite similar to the findings of Nie et al. [45] for Cuba aged 18+. For cardiometabolic risks, the rate of hypertension (16.7%) was much lower than that among U.S. adults (the age-adjusted prevalence of hypertension among the US adults aged 18 and over was 29.1% in 2011/2012 [50]). Approximately 15% of Cubans have high total cholesterol, which is substantially lower than that of Americans, with the prevalence of about 43% [51]. However, about 57% of Cubans have low levels of HDL cholesterol, a higher proportion than that in the US, with a prevalence of around 20% [51]. The findings of the present study contrast sharply with US evidence of Whites having generally more favorable health profiles than other racial/ethnic groups, especially Blacks, who have a higher risk of obesity, hypertension, and diabetes [52, 53]. Although the evidence reported here for Cuba does show people identified as having Black skin colour having a significantly higher probability of hypertension (relative to those identified as having White skin colour), this result contradicts a past study of the urban population of Cienfuegos in central Cuba, which observed no self-assessed skin colour differences in hypertension [54].

The nonexistence of an SEP-health gradient in Cuba could be attributable to the fact that Cubans have benefited from a universal health care system that focuses particularly on primary care and preventive medicine [55], especially given the substantial increase in coverage rate from 61.2% in 1979 to 75.9% in 1989 [56] to 98.2% in 2009 [57]. Its absence could also be influenced by a very well-developed educational system and a generally inclusive society in which racial discrimination is less of an issue than in many other countries.

Admittedly the current study was limited by uncertainties of the income variable (43% of values missing from the data), which led to a substantial difference between the NSRFCD-based average monthly salary of around 227 CUP and the official 2010 number of 448 CUP [58]. In fact, in one assessment of Nie et al. [45] the extent to which the NSRFCD income data follow a Benford distribution [59] indicated clear rejection of this assumption with a chi-square of 2775, whose very large magnitude itself raises serious concerns about data reliability. Nonetheless, the NSRFCD is not only the most recent nationally representative dataset for Cuba that contains detailed information on health outcomes, but one that employs vigorous quality assurance and control procedures.

The current investigation was also at times hindered by relatively small sample sizes (e.g. those for HDL cholesterol and composite biomarkers), which limited the explanatory power of the models. It was, however, the first attempt to explore the SEP-health gradient and its underlying mechanisms in Cuba using both subjective and objective health measures. Two additional limitations were the age of the data and the cross-sectional setting, respectively, which prevented the capture of Cuba’s significant economic changes over the last decade and the drawing of conclusions on causality or temporality [60]. Further studies are thus needed to examine the SEP-health gradients over time using longitudinal data sets.

## Conclusions

Overall, this study offers little evidence of an SEP-health gradient in Cuba, especially as it relates to objective measures of health. Its findings do, however, underscore the importance of incorporating biological indicators, especially for societies experiencing serious health problems that may not always translate into a correlation between self-assessments of personal health and objective health measures.

## Supplementary information

**Additional file 1: Table A1.** Summary of SES and health gradients among Cuban aged 18–65. **Table A2.** OLS/linear probability estimates in SRH, obesity and hypertension among Cuban aged 18–65: NSRFCD 2010. **Table A3.** Linear probability estimates in biomarkers among Cuban aged 18–65: NSRFCD 2010. **Table A4.** Linear probability estimates in composite health indicators among Cuban aged 18–65: NSRFCD 2010

## Data Availability

The datasets used and analyzed during the current study are available from the corresponding author on reasonable request.

## Abbreviations

BMI: Body mass index
CVD: Cardiovascular disease
CUP: Cuban pesos
GDP: Gross domestic product
HDL: High-density lipoprotein
LMICs: Low and middle-income countries
LPMs: Linear probability models
NCDs: Non-communicable chronic diseases
NSRFCD: National Survey on Risk Factors and Chronic Diseases
OLS: Ordinary least squares
PA: Physical activity
SEP: Socioeconomic position
SRH: Self-reported health
SA: Sedentary activity
WC: Waist circumference
