# Supplementary material for: Socioeconomic position and the health gradient in Cuba: dimensions and mechanisms
Source: BMC Public Health. 2020 Jun 5;20:866. doi: 10.1186/s12889-020-08980-3 (PMC7275493; doi:10.1186/s12889-020-08980-3)
Supplement: Supplementary file 1 — Additional file 1: Table A1. Summary of SES and health gradients among Cuban aged 18–65. Table A2. OLS/linear probability estimates in SRH, obesity and hypertension among Cuban aged 18–65: NSRFCD 2010. Table A3. Linear probability estimates in biomarkers among Cuban aged 18–65: NSRFCD 2010. Table A4. Linear probability estimates in composite health indicators among Cuban aged 18–65: NSRFCD 2010 [file 12889_2020_8980_MOESM1_ESM.docx]

**Table A1** Summary of SES and health gradients among Cuban aged 18-65

|  | **SES Dimension** | | | | | | |
| --- | --- | --- | --- | --- | --- | --- | --- |
| **Health outcome** | **Education (Ref. = low)** | | **Occupation (Ref. = unemployed)** | | | **Skin colour (Ref. = White)** | |
|  | Middle | High | Housewife  /houseman | Self-employed | State worker | Mulatto/ Mestizo | Black |
| 1. SRH | N=4,124 | | | | | | |
| Model 1 | Sig. & Pos. | Sig. & Pos. | No sig. | No sig. | No sig. | No sig. | No sig. |
| Model 2 | No sig. | Sig. & Pos. | No sig. | No sig. | No sig. | No sig. | No sig. |
| Model 3 | Sig. & Pos. | Sig. & Pos. | No sig. | No sig. | No sig. | No sig. | No sig. |
| Model 4 | Sig. & Pos. | Sig. & Pos. | No sig. | No sig. | No sig. | No sig. | No sig. |
| Model 5 | No sig. | Sig. & Pos. | No sig. | No sig. | No sig. | No sig. | No sig. |
| 2. Hypertension | N=3,757 | | | | | | |
| Model 1 | No sig. | No sig. | No sig. | No sig. | No sig. | No sig. | Sig. & Pos. |
| Model 2 | No sig. | No sig. | No sig. | No sig. | No sig. | No sig. | Sig. & Pos. |
| Model 3 | No sig. | No sig. | No sig. | No sig. | No sig. | No sig. | No sig. |
| Model 4 | No sig. | No sig. | No sig. | No sig. | No sig. | No sig. | No sig. |
| Model 5 | No sig. | No sig. | No sig. | No sig. | No sig. | No sig. | No sig. |
| 3. General obesity | N=3,741 | | | | | | |
| Model 1 | No sig. | No sig. | No sig. | No sig. | No sig. | No sig. | No sig. |
| Model 2 | No sig. | No sig. | No sig. | No sig. | No sig. | No sig. | No sig. |
| Model 3 | No sig. | No sig. | No sig. | No sig. | No sig. | No sig. | No sig. |
| Model 4 | No sig. | No sig. | No sig. | No sig. | No sig. | No sig. | No sig. |
| Model 5 | No sig. | No sig. | Sig. & Pos. | No sig. | No sig. | No sig. | No sig. |
| 4. Abdominal obesity | N=3,764 | | | | | | |
| Model 1 | No sig. | No sig. | No sig. | No sig. | No sig. | No sig. | No sig. |
| Model 2 | No sig. | No sig. | No sig. | No sig. | No sig. | No sig. | No sig. |
| Model 3 | No sig. | No sig. | No sig. | No sig. | No sig. | No sig. | No sig. |
| Model 4 | No sig. | No sig. | No sig. | No sig. | No sig. | No sig. | No sig. |
| Model 5 | No sig. | No sig. | No sig. | No sig. | No sig. | No sig. | No sig. |
| 5. High glucose | N=1,009 | | | | | | |
| Model 1 | No sig. | No sig. | No sig. | No sig. | No sig. | No sig. | No sig. |
| Model 2 | No sig. | No sig. | No sig. | No sig. | No sig. | No sig. | No sig. |
| Model 3 | No sig. | No sig. | No sig. | No sig. | No sig. | No sig. | No sig. |
| Model 4 | No sig. | No sig. | No sig. | No sig. | Sig. & Pos. | No sig. | No sig. |
| Model 5 | No sig. | No sig. | No sig. | No sig. | No sig. | No sig. | No sig. |
| 6. High cholesterol | N=1,018 | | | | | | |
| Model 1 | No sig. | No sig. | No sig. | No sig. | No sig. | Sig. & Neg. | No sig. |
| Model 2 | No sig. | No sig. | No sig. | No sig. | No sig. | Sig. & Neg. | No sig. |
| Model 3 | No sig. | No sig. | No sig. | No sig. | No sig. | Sig. & Neg. | No sig. |
| Model 4 | No sig. | No sig. | No sig. | No sig. | No sig. | Sig. & Neg. | No sig. |
| Model 5 | No sig. | No sig. | No sig. | No sig. | No sig. | Sig. & Neg. | No sig. |
| 7. High triglycerides | N=1,008 | | | | | | |
| Model 1 | No sig. | No sig. | No sig. | No sig. | No sig. | No sig. | No sig. |
| Model 2 | No sig. | No sig. | No sig. | No sig. | No sig. | No sig. | No sig. |
| Model 3 | No sig. | No sig. | No sig. | No sig. | No sig. | No sig. | No sig. |
| Model 4 | No sig. | No sig. | No sig. | No sig. | No sig. | No sig. | No sig. |
| Model 5 | No sig. | No sig. | No sig. | No sig. | No sig. | No sig. | No sig. |
| 8. Low HDL cholesterol | N=246 | | | | | | |
| Model 1 | No sig. | No sig. | No sig. | No sig. | No sig. | No sig. | No sig. |
| Model 2 | No sig. | No sig. | No sig. | No sig. | No sig. | No sig. | No sig. |
| Model 3 | No sig. | No sig. | No sig. | No sig. | No sig. | No sig. | No sig. |
| Model 4 | No sig. | No sig. | No sig. | No sig. | No sig. | No sig. | No sig. |
| Model 5 | No sig. | No sig. | No sig. | No sig. | No sig. | No sig. | No sig. |
| 9. Metabolic syndrome | N=244 | | | | | | |
| Model 1 | No sig. | No sig. | No sig. | No sig. | No sig. | No sig. | No sig. |
| Model 2 | No sig. | No sig. | No sig. | No sig. | No sig. | No sig. | No sig. |
| Model 3 | No sig. | No sig. | No sig. | No sig. | No sig. | No sig. | No sig. |
| Model 4 | No sig. | No sig. | No sig. | No sig. | No sig. | No sig. | No sig. |
| Model 5 | No sig. | No sig. | No sig. | No sig. | No sig. | No sig. | No sig. |
| 10. One CVD risk factor | N=234 | | | | | | |
| Model 1 | No sig. | No sig. | No sig. | No sig. | No sig. | No sig. | No sig. |
| Model 2 | No sig. | No sig. | No sig. | No sig. | No sig. | No sig. | No sig. |
| Model 3 | No sig. | No sig. | No sig. | No sig. | No sig. | No sig. | No sig. |
| Model 4 | No sig. | No sig. | No sig. | No sig. | No sig. | No sig. | No sig. |
| Model 5 | No sig. | No sig. | No sig. | No sig. | No sig. | No sig. | No sig. |

*Notes*: Sig. & Pos. = significant (p<0.01) and positive association, Sig. & Neg. = significant (p<0.01) and negative association, No sig. = no significant (p<0.01) association. Model 1: OLS/LPM regressions adjusted for gender, age group, marital status, region and provincial dummies; Model 2: Model 1 + other SESs; Model 3: Model 1 + behavioral variables; Model 4: Model 1 + risk perceptions; Model 5: Model 1 + other SESs + behavioral variables + risk perceptions.

**Table A2** OLS/linear probability estimates in SRH, obesity and hypertension among Cuban aged 18-65: NSRFCD 2010

|  | (1) | (2) | (3) | (4) |
| --- | --- | --- | --- | --- |
|  | **Panel A: SRH (N=2,441)** | | | |
| Income | 1.986* | 1.792 | 1.825 | 1.678 |
| 95% CIs | [0.635,3.337] | [0.363,3.221] | [0.444,3.206] | [0.253,3.103] |
|  | **Panel B: General obesity (N=2,221)** | | | |
| Income | 0.013 | 0.012 | 0.012 | 0.011 |
| 95% CIs | [-0.017,0.043] | [-0.021,0.044] | [-0.021,0.046] | [-0.025,0.047] |
|  | **Panel C: Abdominal obesity (N=2,234)** | | | |
| Income | 0.020 | 0.018 | 0.016 | 0.015 |
| 95% CIs | [-0.010,0.050] | [-0.012,0.048] | [-0.011,0.044] | [-0.013,0.043] |
|  | **Panel D: Hypertension (N=2,229)** | | | |
| Income | 0.004 | 0.002 | 0.004 | 0.003 |
| 95% CIs | [-0.020,0.028] | [-0.022,0.027] | [-0.021,0.029] | [-0.024,0.029] |
| Demographic variables | √ | √ | √ | √ |
| Behavioural variables |  | √ |  | √ |
| Risk perception |  |  | √ | √ |

*Notes:* Sample weights are applied. * p < 0.01.

**Table A3** Linear probability estimates in biomarkers among Cuban aged 18-65: NSRFCD 2010

|  | (1) | (2) | (3) | (4) |
| --- | --- | --- | --- | --- |
|  | **Panel A: High glucose (N=600)** | | | |
| Income | 0.009 | 0.007 | 0.014 | 0.010 |
| 95% CIs | [-0.010,0.028] | [-0.008,0.022] | [-0.010,0.038] | [-0.009,0.028] |
|  | **Panel B: High total cholesterol (N=610)** | | | |
| Income | -0.009 | 0.003 | -0.001 | 0.011 |
| 95% CIs | [-0.051,0.034] | [-0.041,0.047] | [-0.038,0.036] | [-0.032,0.053] |
|  | **Panel C: Low HDL cholesterol (N=168)** | | | |
| Income | -0.142 | -0.140 | -0.205 | -0.222 |
| 95% CIs | [-0.323,0.038] | [-0.328,0.049] | [-0.358,-0.052] | [-0.372,-0.071] |
|  | **Panel D: High triglycerides (N=598)** | | | |
| Income | -0.020 | -0.035 | -0.028 | -0.040 |
| 95% CIs | [-0.050,0.010] | [-0.072,0.002] | [-0.056,-0.0003] | [-0.070,-0.010] |
| Demographic variables | √ | √ | √ | √ |
| Behavioural variables |  | √ |  | √ |
| Risk perception |  |  | √ | √ |

*Notes:* Sample weights are applied. * p < 0.01.

**Table A4** Linear probability estimates in composite health indicators among Cuban aged 18-65: NSRFCD 2010

|  | (1) | (2) | (3) | (4) |
| --- | --- | --- | --- | --- |
|  | **Panel A: Metabolic syndrome (N=167)** | | | |
| Income | -0.012 | -0.046 | -0.008 | -0.023 |
| 95% CIs | [-0.078,0.053] | [-0.120,0.029] | [-0.028,0.011] | [-0.102,0.056] |
|  | **Panel B: One CVD risk factor (N=160)** | | | |
| Income | -0.068 | -0.058 | -0.002 | 0.024 |
| 95% CIs | [-0.144,0.007] | [-0.078,-0.039] | [-0.028,0.023] | [-0.123,0.172] |
| Demographic variables | √ | √ | √ | √ |
| Behavioural variables |  | √ |  | √ |
| Risk perception |  |  | √ | √ |

*Notes:* Sample weights are applied. * p < 0.01.
